# Effects of European authorised level of zinc from different sources on the physiology and intestinal ecosystem and performance of piglets weaned at different ages

**DOI:** 10.1016/j.vas.2025.100518

**Published:** 2025-10-10

**Authors:** C. Negrini, D. Luise, F. Correa, M. Mazzoni, A. Serra, A. Monteiro, P. Trevisi

**Affiliations:** a Department of Agricultural and Food Sciences (DISTAL), University of Bologna, Viale G. Fanin 46, 40127 Bologna, Italy; b Department of Veterinary Science, University of Bologna, 46, 40064, Ozzano dell’Emilia, Italy; c Department of Agriculture, Food and Environment, University of Pisa, Via del Borghetto 80, 56124 Pisa, Italy; d Animine, 10 rue Léon Rey Grange, 74960 Annecy, France

**Keywords:** Early weaning, Gut health, Lactobacillaceae, Jejunum morphology, Zinc source

## Abstract

Early weaning can impact piglets’ gut health, and some forms of zinc (Zn) may mitigate this stress. This study evaluates the effect of authorised level of Zn, from different sources, on the performance and gut health of piglets weaned at different ages. At weaning (d0), 96 piglets weaned at either 21 (Early, E; 48) or 26 (Normal, N; 48) days were divided into 3 feeding groups: Zn sulphate (SO_4_), Zn glycinate (Gly) or a porous form of Zn oxide (Hi) to achieve 150 mg/kg of Zn. On d8 and d21, 8 piglets/group were slaughtered and oxidative status (blood), jejunal morphology, gene expression, microbiota, and volatile fatty acids and intestinal pH were assessed. The N group had a higher body weight throughout the study (*P* < 0.001) and a higher gain to feed ratio from d7-d14 (*P* = 0.03). At d8, villus height and villus height:crypt depth ratio (VH:CD) tended to be higher in the N group (*P* < 0.10). At d8, the Hi diet increased the abundance of the Lactobacillaceae (*P* = 0.04) in the E piglets. At d21, the Hi tended to increase VH:CD (*P* = 0.09) compared to SO_4_ in N piglets; in the E groups fed SO_4_ the crypt depth tended to be higher than Gly and Hi (*P* < 0.10). Regardless the weaning age, the Gly tended to increase the expression of Glutathione Peroxidase-2 (*P* = 0.09) and Hi reduced the one of Solute Carrier Family-39 Member-4 (*P* = 0.07). Weaning age impacted piglets’ performance and gut physiology; feeding piglets different forms of Zn may positively improve gut health depending on weaning age.

## Highlights

• Early weaning can lead to developmental and digestive problems in weaned piglets.
• Piglets weaned later can contribute to improve growth performance.
• Porous ZnO reduces gut damage and promoted gut ecosystem in early weaned pigs.

## 1 Introduction

Piglets’ weaning period is characterised by significant and abrupt physiological, environmental and social challenges that can increase the risk of disease and production losses. If a piglet is unable to cope effectively with these weaning stressors, it may experience reduced feed intake (FI), leading to increased gut permeability and subsequent health problems (Campbell et al., 2013). Therefore, the occurrence of post-weaning diarrhoea in newly weaned piglets is common, resulting in large economic losses due to piglets’ morbidity, mortality and treatment costs (Holman et al., 2021).

The weaning age, which can vary between production systems, can significantly affect the piglet’s capability to cope with the weaning stressors. According to European legislation, piglets cannot be weaned before 28 days old unless for welfare or health reasons which reduce the minimum age to 21 days, together with the moving of piglets to a specific infrastructure (European Union Council, 2008). Early weaning (before 21 days) allow to increase the sows' reproductive efficiency, compared to later weaning (25–35 days) (Knox et al., 2013) but it can also heighten digestive health challenges for piglets with underdeveloped digestive and immune systems (Smith et al., 2010; Moeser et al., 2017). Indeed, the greater intestinal immaturity of early weaned piglets, combined with the interruption in the supply of immunoglobulins and other components from the milk, contributes to an increased susceptibility to pathogens, increasing piglet’s disorders and diseases (Cao et al., 2019; St-Pierre et al., 2023). It is known that piglets weaned between 14 and 23 days of age have significantly worse growth performance than piglets weaned between 28 and 35 days of age (Do, 2012; McLamb et al., 2013). Recent studies suggest that there is a linear increase in the growth performance of weaned piglets as the weaning age increases up to 25 days, which appears to be the optimal age for weaning (Faccin et al., 2020a, 2020b).

Among the nutrients essential for proper growth and development during this early stage, zinc (Zn) plays a particularly critical role. Indeed, it is a key component of enzymes involved in the metabolism of carbohydrates, proteins, and fats (Holodova et al., 2019), it is vital for bone formation, immune function, and various metabolic processes (Bonaventura et al., 2015). This trace element is also known to play an important role in supporting the immune system, being involved in the functioning of immune cells, and assisting in the response to infection and diseases (Wessels et al., 2021). Therefore, for a piglets’ proper growth and development, especially in the early stages of life, adequate level as well as form of Zn is essential (Hostetler et al., 2003). Indeed, the supplementation of the piglets’ diet with additional Zn is a standard method used to meet the nutritional requirements.

Nowadays, Zn is available in different forms and sources including chelated, inorganic, coated, porous and nanoparticles Zn, which can have different effects on piglet’s growth and gut health as reviewed by Luise et al. (2024). Chelated Zn forms refers to the binding of the mineral to organic molecules, such as amino acids or proteins (Byrne & Murphy, 2022). Chelated sources can exhibit varying solubilities (e.g., water-soluble and water-insoluble forms) and different stabilities across pH ranges, as has been demonstrated for chelated copper sources (Byrne & Murphy, 2022). In contrast, sulphates and oxides are well-characterised sources, with sulphates being water-soluble and oxides being water-insoluble. Chelates are believed to have higher bioavailability (defined as the proportion of the nutrient that is absorbed and utilized by the body) compared to inorganic sources like standard zinc oxide (ZnO) or Zn sulphate (ZnSO_4_) (Zhang et al., 2018). In piglets, chelate Zn sources seems to have higher digestibility and availability for metabolic and immune processes (Ao et al., 2009; Feng et al., 2010; Liu et al., 2020). Recently, a porous form of ZnO (HiZox®; Hi), characterised by a higher surface area and bigger aggregates, have demonstrated higher bioavailability than standard ZnO sources (Cardoso et al., 2021). When compared to a chelated source (Zn glycinate), a recent study showed that supplementing piglets with 100 mg of Zn/kg resulted in higher Zn apparent total tract digestibility for porous ZnO (Nielsen et al., 2022). This porous form of ZnO also demonstrated to improve gut integrity in weaning piglets (Wang et al., 2019), especially in low birth weight piglets (Negrini et al., 2025), which indicate that this form might be more effective than ZnSO_4_ and equally effective than Zn glycinate form of Zn in maintaining the gut health of early post-weaning piglets. Therefore, the present study hypothesises that Zn sources with differing solubility and bioavailability, may exert distinct effects on the gut health of weaned piglets, with these varying according to the piglets’ stage of intestinal maturation. The aim of this study was to assess the impact authorised level of Zn from either ZnSO₄, Zn-Gly, and Hi forms on performance, health status, intestinal mucosal morphology, and inflammatory status in piglets weaned at either 21 or 26 days of age.

## 2 Material and methods

The in vivo trial was approved by the Ethics Committee for Experiments on Animals of the University of Bologna, Italy and by the Italian Ministry of Health (ID: 4401, 2216A.N.MCL, released 16th December 2022 in compliance with art. 31 of the D.lgs. 26/2014) and complied with the Animal Research Reporting of In Vivo Experiments (ARRIVE) guidelines (Percie du Sert et al., 2020).

### 2.1 Experimental design

A total of 96 piglets were selected from 13 multiparous sows (from 2nd to 8th parity) in an Italian commercial farm (Northern Italy). The study was conducted in two consecutive batches of 48 piglets each. In each batch, the piglets were divided in normal (26 days at weaning; average body weight (BW): 9160 ± 66 g) weaning age and early (21 days at weaning; average BW: 6753.75 ± 37 g). At weaning (d0), the piglets were moved from the commercial farm to the experimental unit of the University of Bologna and divided into six experimental groups following a 2 × 3 factorial design: the first factor was the weaning age: normal weaning age (N) or early weaning age (E). The second factor was the source of Zn added to a standard weaning diet: 1) Zn from ZnSO_4_ (SO_4_); 2) Zn from Zn-Gly (Gly); 3) Zn from a porous form of Zn (Hi; HiZox®, Animine, France).

Piglets of each group were divided into 8 replicates (2 piglets/replicate) balanced for the litter of origin and weaning BW. The piglets were fed ad libitum in a dry feeder with continuous access to the water. The piglets were housed in pens with a slatted floors containing enrichment materials comprising in cotton rope and metal chain. Room temperature was kept controlled from 30 °C from the first day of the trial to 25 °C at the end of the trial, with a 1 °C decrease every 3 days. Moreover, infrared lamps were kept during the first 10 days post-weaning.

### 2.2 Diets

The diets composition and the calculated nutritional values are reported in Supplementary Table S1. The concentration of the basal Zn was analysed on the initial formula and then additional quotes of ZnSO_4_, Zn-Gly or Hi, were added in order to reach around 150 mg/kg of total Zn in all the diets. The supplemented diets were analysed to check the final concentration of Zn in each experimental diet (Supplementary Table S1). The variability observed in the final Zn doses is in line with the expected analytical variability.

### 2.3 Measurement and sample collection

Piglets were individually weighed at d0 and then weekly until d21 post-weaning (end of the trial). FI of each replicate was daily recorded. General health status and faecal consistency (based on 5 points- scale: 1 hard faeces – 5 watery faeces) of each piglet were daily recorded. When piglets had a faecal score >3, they were considered to have diarrhoea (Correa et al., 2022).

Piglets were slaughtered at two time-points: d8 (post-weaning acute phase), one piglet/replicate (a total of 48 piglets) and d21 (post-weaning recovery phase; 48 piglets). Piglets were anaesthetised with Zoletil 100 (Virbac, Milano, Italy; 15 mg/kg BW) and then sacrificed with an intracardiac injection of 0.5 mL/kg BW of Tanax ® (embutramide 200 mg/m, mebenzonium iodide 50 mg/ml tetracaine hydrochloride 5 mg/ml; Intervet Productions srl, Aprilia, Italy). Blood samples were collected before slaughtering at d8 (sacrificed piglets) and d21 (the remaining piglets) into 10 mL collection tube with clot activator (Vacutest Kima Padova, Italy). Samples were let clotted for two hours and then centrifuged at 2000 x g at room temperature for 10 min to obtain serum. Serum was then transferred to 2 mL microtubes and stored at –80 °C for further reactive oxygen metabolites (ROMs) analysis. Intestinal content from distal jejunum, cecum, and colon was immediately sampled after the slaughter and processed to determine the pH value (Vio, Giorgio Bormac S.r.l., Carpi (MO) Italy). In addition, a sample of jejunum content was collected into a sterile collection tube, immediately frozen in liquid nitrogen, and then stored at −80 °C for microbiota and volatile fatty acids (VFAs) and lactic acid analysis. From each piglet, two samples of distal jejunal mucosa were collected, the first one was immediately frozen in liquid nitrogen and then stored at −80 °C for gene expression analyses, and the second one was fixed in buffered formalin for morphological analyses.

### 2.4 Blood analysis

Concentration of ROMs was detected colorimetrically from the serum samples using the d-ROMs test kit (Diacron International Sr1, Grosseto, Italy) after a dilution of 1:20 with distilled water and incubation for 5 min at 37 °C with mixture containing 0.01 M acetic acid/sodium acetate buffer pH 4.8 and N,N diethyl p-phenylenediamine as chromogen (Brambilla et al., 2002).

### 2.5 Gene expression analysis of jejunum mucosa

Jejunal mucosal total RNA was extracted using the GeneJET RNA Purification Kit (Thermo Fisher Scientific, Waltham, MA, USA) following the manufacturer’s instructions and treated with the TURBO DNA-free™ DNA Removal Kit (Thermo Fisher Scientific, Waltham, MA, USA). After the RNA quality and quantity evaluation a total of 800 µg of RNA was converted into complementary DNA using the High-Capacity RNA-to-cDNA™ Kit (Thermo Fisher Scientific, Waltham, MA, USA) according to the manufacturer’s instructions. Duplex Real Time PCR reactions contained 2 µl cDNA and 8 µl mix containing the Taqman Assay (Supplementary Table S2) and 2X TaqMan Mastermix and run in triplicate on the Applied Biosystems QuantStudio™ 7 Flex Real-Time PCR system (Thermo Fisher Scientific, Waltham, MA, USA). Hydroxymethylbilane synthase (*HMBS*) was used as housekeeping gene. The following genes were selected and analysed: Nuclear Factor Kappa B Subunit 2 (*NFKB2*), Claudin-4 (*CLAUD4*), Glutathione Peroxidase 2 (*GPX-2*), Solute Carrier Family 39 Member 4 (*SLC39A4*) and Solute Carrier Family 30 Member 7 (*SLC30A7*). The latter two genes were selected to assess the Zn transport in the small intestine of piglets. The expression of the target gene was given as fold change calculated by 2-ΔΔ Ct.

### 2.6 Morphological analysis of jejunum mucosa

For morphological analyses, the jejunum samples were fixed for 48 h in 10 % buffered formalin and embedded in paraffin. Paraffin thick sections (7 µm) were stained with haematoxylin-eosin for morphological evaluation. Analyses were carried out as previously described by Luise et al. (2023) on fifteen villi and fifteen crypts from each sample were analysed.

### 2.7 Bacterial DNA extraction and bioinformatic analysis from jejunum content

For the microbiota analysis, bacterial DNA extraction from jejunum content was carried out using HostZERO™ Microbial DNA Kit (Zymo Research, Orange, CA, USA) following the manufacturer’s instructions. DNA concentration and purity (absorbance ratio 260/280 and 260/230) of the isolated DNA were checked on the NanoDrop (Fisher Scientific, 13 Schwerte, Germany). The V3-V4 regions of the 16S rRNA gene (∼460 bp) were amplified. Amplicons were produced using the universal primers reported by Takahashi et al. (2014) using the Platinum™ Taq DNA Polymerase High Fidelity (Termo Fisher Scientific, Italy). The libraries were prepared using the standard protocol for MiSeq Reagent Kit V3 and sequenced on Illumina MiSeq platform 300×2 bp (Illumina Inc., San Diego, Ca, USA). For the bioinformatics analysis, as pipeline DADA2 was used (Callahan et al., 2016) taking in consideration the Silva database (Quast et al., 2013) (version 138.1) as reference for the taxonomic assignment. Prior analyses, the primers were removed from the raw sequences, and forward and reverse reads were cut at positions 290 and 250 based on the average quality score.

### 2.8 Volatile fatty acids and lactic acid analysis of jejunum content

The analysis of VFA (acetate, propionate; isobutyrate; butyrate; valerate, isovalerate) and lactic acid of jejunum content samples was performed by High Performance Liquid Chromatography (HPLC) according to the following procedure described by Trevisi et al. (2023). Quantification was done using an external calibration curve based on the standards described by Trevisi et al. (2023).

### 2.9 Statistical analysis

Statistical analyses were carried out in R v3.6 environment using “lsmeans” (Lenth, 2021), “car” (Fox & Weisberg, 2019), and “lm4” (Bates et al., 2015) packages. Data collected at individual level were analysed using a linear mixed model followed by the ANOVA procedure in which the diet, weaning age, batch and the interaction between diet and weaning age were included as fixed factors and litter of origin was included as a random factor. Data collected at pen level were analysed using a linear and ANOVA model including the diet, weaning age and their interaction as fixed factors. To explore the effect of dietary Zn sources within each class of weaning age, pairwise contrasts between dietary treatments were performed separately within each class of weaning age, with Tukey’s adjustment applied for multiple comparisons. For data that did not follow a normal distribution (VFAs), the Box-Cox normalisation was performed prior to the analysis. The batch was removed when not significant.

Furthermore, since some results on the growth performance could be attributed merely to the different physiology of N and E piglets, performance data of the E group were shifted one week forward to reduce the differences in term of age with N piglets and the statistical analyses were carried out as mentioned beforehand.

Regarding the microbial data, the statistical analyses on alpha diversity, beta diversity and taxonomic composition were performed with R software using the vegan 2.6–4 (Dixon, 2003), phyloseq 1.44.0 (McMurdie & Holmes, 2011), and microbiomeMarker 1.6 (Cao et al., 2022) packages. Prior to the statistical analyses, abundance data were rarefied to the lowest sampling depth. Chao1, Shannon, and Simpson diversity indices were calculated and analysed using a linear model followed by ANOVA procedure including diet, weaning age, and their interaction as factors for the alpha diversity. Same contrasts used for the other data were performed. For the beta diversity, the Bray Curtis distance matrix was calculated and plotted using a Nonmetric multidimensional scaling (NMDS) plot. The effects of diets, weaning age, and their interaction were tested using a non-parametric PERMANOVA model (Adonis test), with 999 permutations. The differential abundance analysis on the different taxa was performed using Linear discriminant analysis Effect Size (LEfSe) (Segata et al., 2011) implemented in the wrapper function included in the package microbiomeMarker 1.6 at the amplicon sequence variants (ASVs). A cut-off of 3 and P.adj<0.05 were considered for the LEfSe analysis.

A significant effect was declared at *P* < 0.05, and *P* ≥ 0.05 and ≤ 0.10 was considered a tendency.

## 3 Results

### 3.1 Health and performance

Table 1 reports the effect of diet, weaning age and their interaction on the growth performance results. BW was not affected by the diet nor the interaction between the weaning age and the diet. The weaning age affected the BW through the entire trial (*P* < 0.001), resulting higher in the N piglets. ADG was not affected by the diet nor the interaction between the weaning age and the diet. The weaning age affected ADG for the periods: d7-d14, d7-d21 and d0-d21, (*P* = 0.02, *P* = 0.01, *P* = 0.04 respectively) and tended to influence ADG for the periods d0-d14 and d14-d21, (*P* = 0.06, *P* = 0.08, respectively). In all periods, N piglets had higher ADG compared to the E piglets. A trend of significance was observed for the interaction between diet and weaning age in the FI of the period d0-d7 (*P* = 0.07). The diet never affected FI. The weaning age influenced the FI for the periods d7-d14 (*P* = 0.003), d0-d14 (*P* = 0.004), d0-d21 (*P* = 0.005), d7-d21 (*P* = 0.005), d14-d21 (*P* = 0.02) and it was higher in the N group compared to the E group for all the periods. The interaction between diet and weaning tended to affect the G:F ratio at d7-d21 (*P* = 0.07). The G:F ratio was not affected by the diet nor the weaning age.

**Table 1:** Effect of source of dietary zinc, weaning age and their interaction on the performance parameters of post-weaning piglets weaned at different age.

Item	Early			SEM	Normal			SEM	P-value		
	SO_4_	Gly	Hi		SO_4_	Gly	Hi		Diet^1^	Age^2^	Diet*age
Body weight, g											
d0	6666	6665	6695	214	8621	8613	8621	213	0.99	<0.001	0.99
d7	6918	7137	7001	230	9242	8884	9017	245	0.63	<0.001	0.23
d14	8402	8787	8269	508	11,776	11,314	11,171	537	0.56	<0.001	0.50
d21	10,973	11,340	11,054	788	15,143	15,035	14,734	836	0.88	<0.001	0.89
Average daily gain, g/day											
d0-d7	35.4	66.9	43.6	18	58.2	40.4	59.7	19.1	0.19	0.26	0.12
d7-d14	215	233	206	45.9	328	344	323	49.2	0.83	0.02	0.99
d0-d14	128	153	125	33.1	195	192	185	35.5	0.64	0.06	0.82
d14-d21	371	364	396	57.6	477	531	512	61.9	0.83	0.08	0.71
d7-d21	292	299	302	42.7	404	436	416	46	0.97	0.01	0.89
d0-d21	208	223	216	35.8	290	305	293	38.4	0.90	0.04	0.99
Feed intake, g/day											
d0-d7	90.6	117.4	97.2	14.9	114.8	97.8	119.9	16.1	0.17	0.13	0.07
d7-d14	296	339	295	40.5	423	436	388	43.7	0.46	0.003	0.82
d0-d14	193	228	196	24.7	269	267	254	26.7	0.29	0.004	0.59
d14-d21	502	511	512	77	697	714	705	83.2	0.97	0.02	0.99
d7-d21	399	425	403	53.6	560	575	547	57.9	0.88	0.005	0.97
d0-d21	296	322	301	38	412	416	404	41	0.77	0.005	0.93
Gain to Feed											
d0-d7	0.39	0.51	0.41	0.19	0.50	0.18	0.49	0.21	0.79	0.61	0.19
d7-d14	0.72	0.68	0.69	0.08	0.75	0.79	0.82	0.08	0.93	0.71	0.64
d0-d14	0.66	0.64	0.62	0.08	0.68	0.72	0.72	0.08	0.87	0.82	0.79
d14-d21	0.75	0.70	0.77	0.05	0.68	0.76	0.73	0.05	0.31	0.20	0.17
d7-d21	0.73	0.69	0.75	0.03	0.71	0.78	0.76	0.04	0.20	0.54	0.07
d0-d21	0.71	0.67	0.71	0.04	0.69	0.75	0.72	0.04	0.38	0.59	0.17

1 SO_4_: ZnSO_4_, Gly: Zn-Glycinate, Hi: porous form of ZnO;

2 Early: 21 days at weaning (6753.75 ± 37 g), Normal: 26 days at weaning (9160 ± 66 g).

Supplementary Table 3 shows the results of the effect of weaning age and diet on the growth performance of the piglets considering the same age. Diet did not affect BW, ADG, FI and G:F. Weaning age affected the BW when animals were 26–28 days of age (*P* < 0.0001) and tended to influence it when the piglets had 33–35 days of age (*P* = 0.06), resulting in higher BW in N piglets. The E piglets had a higher ADG (*P* < 0.0001), FI (*P* < 0.01) and G:F (*P* < 0.05) in the period from 26–28 to 33–35 days of age. No differences were observed in the period between 33–35 to 40–42.

Table 2 shows the effect of the diet and weaning age and their interaction on the faecal score. The consistency of the faeces never reached the cut-off of diarrhoea (>3), confirming the piglets’ good health status. The interaction between the diet and weaning age significantly affected the faecal score at d7-d14 (*P* = 0.03), d7-d21 (*P* = 0.02), and d0-d21 (*P* = 0.02), and tended to affect it from d0 to d14 (*P* = 0.07). In period d7-d14, in the piglets of the E group, the SO_4_ and Gly groups tended to have lower faecal score compared to the Hi group (*P* = 0.08). In period d7-d21, in the piglets of the E group, the SO_4_ group tended to have lower faecal score compared to the Hi group (*P* = 0.06).

**Table 2:** Effect of source of dietary zinc, weaning age and their interaction on faecal score of post-weaning piglets weaned at different age.

Item	Early			SEM	Normal			SEM	P-value		
	SO_4_	Gly	Hi		SO_4_	Gly	Hi		Diet^1^	Age^2^	Diet*age
Faecal score											
d0-d7	2.16	2.14	2.08	0.08	2.16	2.08	2.17	0.08	0.55	0.98	0.40
d7-d14	2.06 y	2.04^y^	2.32^x^	0.12	2.25	2.04	2.05	0.13	0.03	0.11	0.03
d0-d14	2.07	2.13	2.23	0.09	2.25	2.06	2.1	0.1	0.22	0.07	0.07
d14-d21	2.00	2.07	2.08	0.06	2.06	2.00	2.00	0.06	0.34	0.32	0.19
d7-d21	2.03 y	2.06 y^x^	2.20 x	0.08	2.17	2.02	2.03	0.08	0.05	0.07	0.02
d0-d21	2.05	2.11	2.17	0.07	2.19	2.04	2.07	0.08	0.16	0.05	0.02

1 SO_4_: ZnSO_4_, Gly: Zn-Glycinate, Hi: porous form of ZnO;

2 Early: 21 days at weaning (6753.75 ± 37 g), Normal: 26 days at weaning (9160 ± 66 g).

xy Means in the same row with different uppercase letters indicate significant differences at >0.05 *P* < 0.10.

### 3.2 Blood parameters

**Fig. 1:**
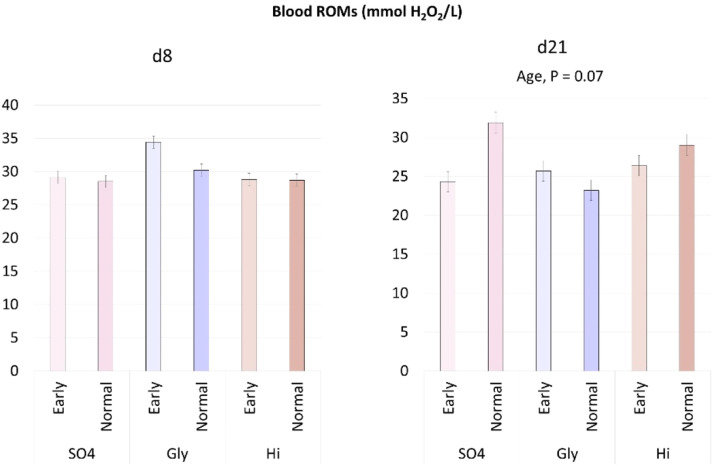
Effect of source of dietary zinc, weaning age and their interaction on the blood ROMs concentration of post-weaning piglets weaned at different age. SO_4_: ZnSO_4_, Gly: Zn-Glycinate, Hi: porous form of ZnO; Early: 21 days at weaning (6753.75 ± 37 g), Normal: 26 days at weaning (9160 ± 66 g).

Fig. 1 shows the effect of the diet, weaning age and their interaction on ROMs concentration at d8 and d21. The interaction between diet and weaning age and the diet were not significant. The weaning age tended to affect ROMs concentrations at d21 (*P* = 0.07), resulting in lower values in the E compared to N piglets.

### 3.3 Jejunal microbial profile

A total of 3 662 661 raw reads which were attributed to a total of 1630 ASVs were obtained. The ASVs were associates to 16 phyla, 110 families and 268 genera. The most abundant phyla belonged to Firmicutes 93 ± 0.12 %, Actinobacteriota 6 ± 0.11 %, and Proteobacteria 1 ± 0.03 %; the most abundant families were Lactobacillaceae 70 ± 0.34 %, Streptococcaceae 10 ± 0.18 %, Clostridiaceae 9 ± 0.23 % and Corynebacteriaceae 4 ± 0.09 %; and the most abundant genera were *Lactobacillus* 33 ± 0.23 %, *HT002*, belonging to the Lactobacillaceae family, 29 ± 0.24 % and *Streptococcus* 10 ± 0.18 %.

**Fig. 2:**
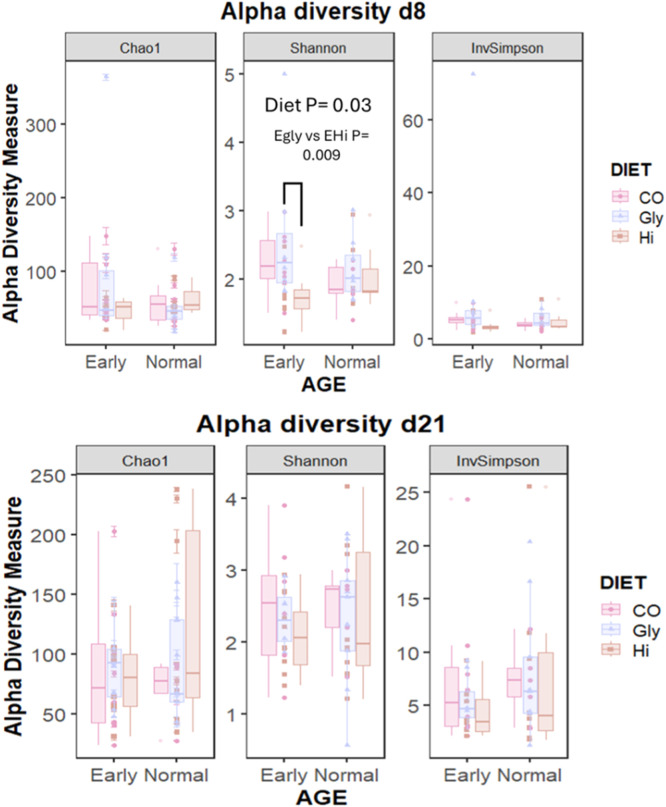
Effect of source of dietary zinc, weaning age and their interaction on alpha diversity indices in jejunum content samples of post-weaning piglets weaned at different age. SO_4_: ZnSO_4_, Gly: Zn-Glycinate, Hi: porous form of ZnO; Early: 21 days at weaning (6753.75 ± 37 g), Normal: 26 days at weaning (9160 ± 66 g).

**Fig. 3:**
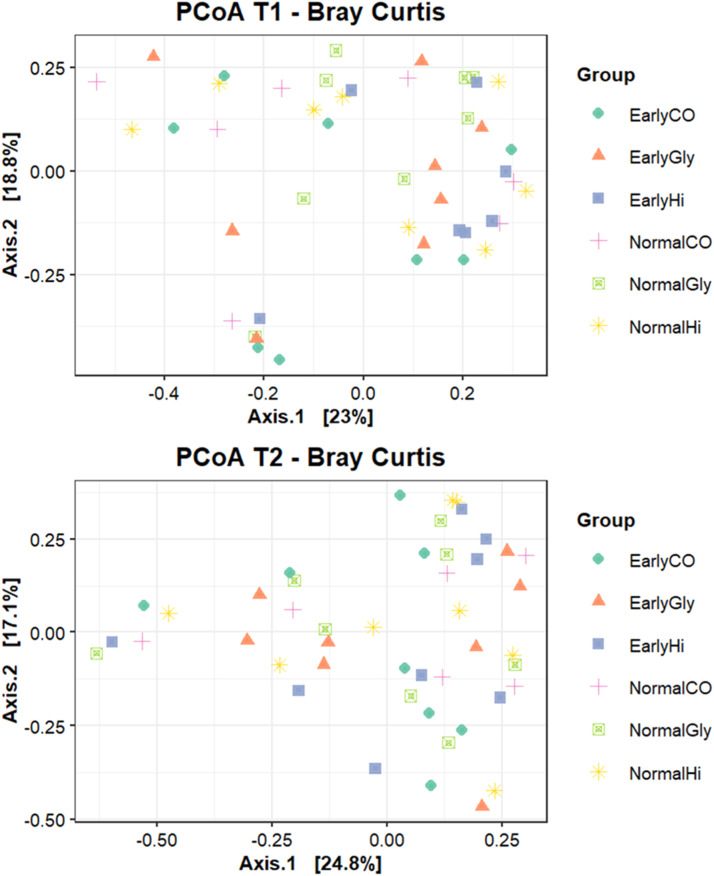
Effect of source of dietary zinc, weaning age and their interaction on beta diversity in jejunum content samples of post-weaning piglets weaned at different age. SO_4_: ZnSO_4_, Gly: Zn-Glycinate, Hi: porous form of ZnO; Early: 21 days at weaning (6753.75 ± 37 g), Normal: 26 days at weaning (9160 ± 66 g).

Alpha and Beta diversity results are shown in Fig. 2, Fig. 3, respectively. Alpha diversity indices were not affected by neither the weaning age and the interaction between diet and weaning age both at d8 and d21. The diet influenced the Shannon index at d8 (*P* = 0.03) and in details, the piglets in the E group fed the Gly had a higher Shannon index compared to the Hi piglets (*P* = 0.02). The other alpha indices at d8 and d21 were not influenced by the diet.

For Beta diversity, the Adonis test did not evidence any effect of the diet, weaning age and their interaction both at d8 and d21.

According to the Lefse analysis, performed at ASVs level, at d8, the piglets in the E group fed the Hi were characterised by a higher abundance of the ASV 24 associated with the genus *HT002* belonging to the Lactobacillaceae family (LDA_score = 4.16, P.adj = 0.04). At d21, the piglets in the E group fed Hi were characterised by a higher abundance of the ASV 43 associated to the genus *Weissella* (LDA_score = 3.57, P.adj = 0.046) (Supplementary Figure S1). No other differences were observed for the other groups at either d8 or d21.

### 3.4 Intestinal contents pH values, volatile fatty acids and lactic acid

Table 3 show the effect of the diet, weaning age and their interaction on the pH of the intestinal contents and the VFAs and lactic acids concentration in the jejunum at d8 and d21.

**Table 3:** Effect of source of dietary zinc, weaning age and their interaction on intestinal content pH and on the volatile fatty acids and lactic acid concentration in the jejunum of post-weaning piglets weaned at different age.

Item	Early			SEM	Normal			SEM	P-value		
	SO_4_	Gly	Hi		SO_4_	Gly	Hi		Diet^1^	Age^2^	Diet*Age
d8											
Jejunum pH	6.71	6.81	6.29	0.25	7.01	6.7	6.85	0.25	0.08	0.24	0.15
Caecum pH	5.85	5.94	5.79	0.23	6.00	6.05	6.13	0.23	0.79	0.51	0.75
Colon pH	6.19	6.24	6.19	0.15	6.26	6.34	6.3	0.16	0.93	0.64	0.99
Lactic acid, (mg/g)	15.6	10	22.3	0.63	22.5	26.5	16.7	0.62	0.13	0.39	0.07
Acetate, (mg/g)	5.46	7.61	5.65	0.35	8.44	7.24	7.36	0.31	0.36	0.12	0.40
Propionate, (mg/g)	1.65	3.9	2.9	0.79	2.55	4.6	1.29	1.92	0.34	0.54	0.25
Iso-butyrate, (mg/g)	1.19	1.14	1.42	0.26	1.57	1.39	1.22	0.36	0.62	0.29	0.44
Butyrate, (mg/g)	2.88	7.63	4.7	1.01	9.39	5.18	4.76	1.26	0.28	0.07	0.18
Valerate, (mg/g)	0.33	0.42	0.29	0.63	0.77	0.65	0.67	0.54	0.66	0.075	0.73
Total, (mg/g)	13.9	22.5	19.4	0.35	31.00	22.5	18.5	0.51	0.24	0.008	0.08
d21											
Jejunum pH	6.91	6.77	6.77	0.22	6.9	6.92	6.97	0.22	0.75	0.97	0.77
Caecum pH	5.74	5.69	5.63	0.18	5.6	5.61	5.71	0.19	0.83	0.47	0.69
Colon pH	5.9	6.02	6.07	0.22	5.97	6.09	5.9	0.24	0.73	0.75	0.68
Lactic acid, (mg/g)	4.96	6.49	5.36	0.41	7.37	6.64	7.82	0.32	0.71	0.29	0.66
Acetate, (mg/g)	10.22	8.47	9.87	0.30	10.85	11.0	12.7	0.26	0.71	0.81	0.81
Propionate, (mg/g)	4.23	4.19	3.01	0.61	7.37	10.3	8.85	0.46	0.59	0.43	0.64
Iso-butyrate, (mg/g)	1.50	1.81	1.48	0.37	2.17	2.01	2.57	0.33	0.74	0.23	0.54
Butyrate, (mg/g)	7.99	2.89	4.71	1.64	9.62	6.58	5.22	1.13	0.20	0.75	0.62
Valerate, (mg/g)	0.69	0.79	0.69	0.46	0.85	0.81	0.77	0.45	0.91	0.63	0.95
Total, (mg/g)	29.0	20.0	25.6	0.36	31.8	32.1	39.1	0.28	0.29	0.71	0.46

1 SO_4_: ZnSO_4_, Gly: Zn-Glycinate, Hi: porous form of ZnO.

2 Early: 21 days at weaning (6753.75 ± 37 g), Normal: 26 days at weaning (9160 ± 66 g).

The pH of the intestinal contents was not affected by the interaction between diet and weaning age and by the weaning age. The diet tended to influence the pH of the jejunum at d8 (*P* = 0.08), but no significant contrasts were observed between the groups.

At d8, the interaction between diet and weaning age did not impact VFAs and lactic acid results, except for the lactic acid concentration (*P* = 0.07) and the total amount of VFAs (*P* = 0.08), but no significant contrasts were observed between the groups. The diet did not affect the results at d8. The weaning age tended to affect butyrate (*P* = 0.07) and valerate concentrations (*P* = 0.075) at d8. Both butyrate and valerate concentration was higher in N piglets compared to E piglets. No effect of diet, weaning age and their interaction were observed at d21.

### 3.5 Jejunum morphology

Table 4 reports the effect of the diet, weaning age and their interaction on the morphological parameters of the jejunum at d8 and d21. The interaction between diet and weaning age did not affect the parameters at d8 and d21.

**Table 4:** Effect of source of dietary zinc, weaning age and their interaction on the jejunum morphology of post-weaning piglets weaned at different age.

Item	Early			SEM	Normal			SEM	P-value		
	SO_4_	Gly	Hi		SO_4_	Gly	Hi		Diet^1^	Age^2^	Diet*age
d8											
Villus height, µm	244	238	270	21.9	293	282	276	22.0	0.29	0.07	0.31
Villus width, µm	80.3	79.8	81.5	3.49	82.3	85	83.4	3.42	0.87	0.78	0.72
Crypt width, µm	33.2	31.5	34.7	1.76	34.1	34	34.2	1.72	0.18	0.80	0.45
Crypt depth; µm	148	145	163	8.78	149	154	152	8.72	0.075	0.93	0.24
M:S ratio^3^	6.67	6.77	6.98	0.51	7.86	7.25	7.3	0.52	0.82	0.04	0.44
VH:CD ratio^4^	1.67	1.73	1.67	0.17	2.00	1.84	1.81	0.17	0.89	0.06	0.57
d21											
Villus height; µm	237	235	244	15.6	232	259	261	16.6	0.80	0.76	0.39
Villus width, µm	71.3^a^	71.8^a^	62.0^b^	3.82	73.7	68.1	67.9	3.99	0.01	0.65	0.17
Crypt width, µm	33.0	28.5	32.4	2.67	33.8	30.5	29.4	2.87	0.17	0.79	0.37
Crypt depth, µm	172.0^x^	149.0^y^	149.0^y^	10.1	160	148	148	10.9	0.02	0.23	0.66
M:S ratio	6.87	7.24	7.5	0.5	6.62	7.95	8.11	0.53	0.42	0.68	0.34
VH:CD ratio	1.39	1.57	1.69	0.15	1.46^y^	1.78^xy^	1.79^x^	0.16	0.09	0.64	0.80

1 SO_4_: ZnSO_4_, Gly: Zn-Glycinate, Hi: porous form of ZnO.

2 Early: 21 days at weaning (6753.75 ± 37 g), Normal: 26 days at weaning (9160 ± 66 g).

3 M:S ratio^3^ : mucosal-to-serosal amplification ratio. Calculated as (villous surface þ unit bottom − villous bottom)/unit bottom, where villous surface = π (villous length × villous width), unit bottom = π (villous width/2 þ crypt width/2)^2^ and villous bottom = π (villous width/2)

4 VH:CD ratio^4^ : villus height/crypt depth.

a,b Means in the same row with different uppercase letters indicate significant differences at *P* < 0.05.

x,y Means in the same row with different uppercase letters indicate significant differences at >0.05 *P* < 0.10.

At d8, the diet tended to affect the crypt depth (*P* = 0.075), but no differences were observed between the groups. At d8, weaning age significantly affected the mucosal-to-serosal amplification ratio (*P* = 0.04) and tended to influence the villus height (*P* = 0.07) and villus height to crypt depth ratio (VH:CD) (*P* = 0.06) with higher values for the N piglets.

At d21, the diet affected the villus width (*P* = 0.01) and crypt depth (*P* = 0.02) and tended to influence the VH:CD ratio (*P* = 0.09). Villus width was higher in piglets in the E group fed SO_4_ (*P* = 0.04) and Gly (*P* = 0.04) compared to Hi. Crypt depth was higher in piglets in the E group fed SO_4_ compared to Gly (*P* = 0.07) and Hi (*P* = 0.06). The VH:CD tended to be higher in piglets of the N group fed Hi compared to SO_4_ (*P* = 0.09).

### 3.6 Jejunal gene expression

Table 5 reports the effect of the diet, weaning age and their interaction on jejunal genes expression at d8 and d21. The interaction between diet and weaning age did not affect the genes’ expression either at d8 and d21.

**Table 5:** Effect of source of dietary zinc, weaning age and their interaction on the jejunum gene expression of post-weaning piglets weaned at different age.

Item	Early			SEM	Normal			SEM	P-value		
	SO_4_	Gly	Hi		SO_4_	Gly	Hi		Diet^1^	Age^2^	Diet*age
d8											
*NFKB2*^3^	0.95	0.93	1.8	0.24	0.82	1.24	1.36	0.24	0.78	0.61	0.31
*Claudin 4*	0.97	1.18	1.29	0.27	1.14	0.87	0.89	0.28	0.46	0.52	0.26
*GPX-2*^4^	1.26	0.81	1.43	0.41	1.57	1.64	1.28	0.42	0.28	0.49	0.22
*SLC39A4*^5^	1.11	1.89	1.04	0.80	1.17	0.74	0.92	0.82	0.48	0.94	0.49
*SLC30A7*^6^	1.17	0.93	1.12	0.36	1.22	0.98	0.28	0.37	0.79	0.89	0.97
d21											
*NFKB2*^3^	1.21	1.07	1.43	0.23	1.03	0.94	1.12	0.26	0.25	0.46	0.83
*Claudin 4*	1.21	0.81	1.06	0.22	1.19	0.86	1.13	0.25	0.18	0.97	0.97
*GPX-2*^4^	0.95	1.63	1.47	0.34	1.17	1.63	0.96	0.38	0.09	0.55	0.29
*SLC39A4*^5^	1.25	1.22	0.61	0.33	1.24	0.77	0.68	0.37	0.07	0.96	0.46
*SLC30A7*^6^	1.05	0.81	1.20	0.23	0.94	0.83	1.12	0.26	0.21	0.66	0.91

1 SO_4_: ZnSO_4_, Gly: Zn-Glycinate, Hi: porous form of ZnO;.

2 Early: 21 days at weaning (6753.75 ± 37 g), Normal: 26 days at weaning (9160 ± 66 g).

3 *NFKB2:* Nuclear Factor Kappa B Subunit 2.

4 *GPX-2:* Glutathione Peroxidase 2.

5 *SLC39A4:* Solute Carrier Family 39 Member 4.

6 *SLC30A7:* Solute Carrier Family 30 Member 7.

At d8, the weaning age and the diet did not affect the genes expression. At d21, the weaning age did not affect any of the tested genes. Diet tended to affect *GPX-2* (*P* = 0.09) and *SLC39A4* (*P* = 0.07) expression. Regardless of the weaning age, the piglets fed Gly tended to have a higher *GPX-2* expression while the piglets fed the SO_4_ diet tended to have a higher *SLC39A4* expression.

## 4 Discussion

The European regulatory framework, as evidenced by the ban on pharmacological doses of Zn, is increasingly highlighting the necessity to improve the sustainability of the swine industry. Only the inclusion of a dose of 150 mg/kg of Zn in piglets’ feed is currently permitted, according to the EMA’s 2017 recommendation (EMA, 2017). Therefore, understanding the differences between the effects of different forms of Zn included in the diet at the European authorized level on piglet growth, health and gut health is crucial.

The results obtained on the growth performance indicated that the different forms of Zn at the European authorised level do not play a crucial role, while the age at weaning was the most impacting factor. Indeed, BW, ADG and FI which were constantly higher in piglets weaned later in agreement with previous studies (Do, 2012; McLamb et al., 2013) and confirming the adequacy of the trial design. Our results evidenced that the observed variations between the two weaning age classes were mainly due to age differences at the time of data recording of piglets and not to a greater weaning discomfort of the E group. In fact, when comparing the weight of E piglets at 42 days of age with the N piglets at 40 days old, there was no difference. Moreover, when we compared the piglets for the actual days of life, we observed that the G:F was higher in the E piglets than in the N piglets in the second week after weaning. Therefore, it can be hypothesised that the piglets grew in accordance with their physiological programme.

Confirming the detrimental effect of early weaning on the gut health of immature piglets, the morphological data indicated a marked stress in the E weaned piglets (Smith et al., 2010; Moeser et al., 2017). Indeed, on day 8, the E group had lower villus height, VH:CD ratio, and absorptive mucosal surface compared to the N piglets, suggesting increased physiological stress related to the early weaning (Smith et al., 2010; Moeser et al., 2017). Furthermore, the E piglets had a lower jejunal concentration of butyrate and valerate at d8. These VFAs are important source of energy for intestinal epithelial cells and can favour the increase in piglet’s growth performance (Cao et al., 2019). These latter result agree with the morphological and performance data observed in the E piglets, confirming the different maturation degree between E and N weaned piglets. Indeed, piglets weaned later may have a milder stress response during the weaning process, which could contribute to better post-weaning performance and overall well-being. Previous data suggested that piglets weaned at 21 days had diminished jejunal morphological characteristics even 14 days post-weaning (Ming et al., 2021). Although no direct comparison can be made between this latter study and the present one, due to the different timing of piglet sacrifice (in the present study we assess it at 21 days post-weaning), it can be hypothesised that the lack of differences in the jejunal morphology between the E and N piglets may be due to the achievement of the small intestinal maturity even in the E weaned piglets. In agreement with the lack of difference in morphological parameters, no difference was observed in VFAs concertation as well as in intestinal pH at d21 and on G:F from d14 to d21.

As already reported in the literature (McCracken et al., 1999; Luise et al., 2024; Negrini et al., 2025), the source of Zn can influence the gut architecture, and gut ecosystem. The three types of Zn used in the present study are hypothesised to differ for their intestinal solubility. In particular, the porous form of ZnO tested in the present study is characterised by a high surface area, leading to a promising comparable result of pharmacological doses of ZnO in terms of growth and gut health, as summarised in the review by Luise et al. (2024). The higher surface area can increase the interaction between the Zn and the microbiota present in the jejunum, indeed, we observed a reduction of the alpha diversity indices (vs Gly), and an increase in the relative abundance of Lactobacillaceae family at d8. The relationship between the use of ZnO and the abundance of intestinal Lactobacillaceae family has already been investigated, with inconsistent results depending on the dose administrated. For instance, the administration of ZnO at high doses (2000 mg/kg) has been associated with a reduction in beneficial bacteria such as *Lactobacillus* (Huynh & Zastrow, 2023; Luise et al., 2024). However, when administrated at lower doses such as 380 and 570 mg/kg, it resulted in an increase in *Lactobacillus* spp. in the faeces of piglets (Peng et al., 2019), which is consistent with the finding observed in the present study.

However, these changes in the intestinal ecosystem obtained with Hi did not translate into improved growth or intestinal morphological parameters of the piglets as we observed no influence of the diet in villus height and VH:CD ratio at d8. In addition, the piglets in the E group receiving the Hi tended to have a higher faecal score from d7 to d14 compared to the Gly and SO_4_ fed piglets, however, it should be considered that the average faecal score was well below the threshold indicative of diarrhoea (faecal score > 3); therefore, it cannot be concluded that this form of Zn adversely affected the intestinal health of the piglets.

The main effects attributable to the Zn sources were observed at d21, during the recovery phase, when both the Hi and Gly reduced the crypt depth in E piglets compared to the SO_4_. Crypt depth is an indicator of epithelial cell development and proliferation, and a reduction in crypt depth is generally associated with improved nutrient absorption efficiency and better intestinal maturation. Previous studies suggested that Zn chelated with amino acid could protect villous epithelial cells and reduce the crypt depth especially in young monogastric animals such as broilers, compared to inorganic form of Zn (Zhu et al., 2022; De Grande et al., 2020). Therefore, the results obtained suggest that even in piglets, at a dose of 150 ppm, the Zn-Gly can contribute to an improved intestinal absorption compared to Zn-SO_4._ In addition, we observed a trend of higher expression of *GPX-2* gene in jejunum of both E and N piglets fed with Gly; *GPX-2* encode for a protein belonging to the glutathione peroxidase family, which contribute to catalyse the reduction of organic hydroperoxides and hydrogen peroxide (H_2_O_2_) by glutathione, and thereby protect cells against oxidative damage. No previous study has investigated this effect of Zn-Gly in the expression of *GPX-2* compared to other Zn sources, however, Zn has been shown to influence the Nrf2 pathway, a key regulator of antioxidant response, leading to the upregulation of genes involved in oxidative stress defence, including *GPX-2* (Prasad & Bao, 2019). Therefore, our results indicate that form of Zn may have a stronger effect on the oxidative status compared to the other two Zn sources.

The administration of Hi, besides decreasing the crypt depth in the E piglets, increased the VH:CD in the N weaned piglets compared to the SO_4_. In accordance with our data, previous studies suggested the positive effect of Hi on intestinal morphological parameters. Indeed, according to Peng et al. (2019), the administration of this porous form of ZnO at both 200 and 500 mg/kg was able to achieve the same villus height and crypt depth as the pharmacological dose of ZnO. More recently, Negrini et al. (2025), observed that Hi was able to reduce the gap in the jejunal morphological parameters between low and normal birth body weight at 21 days post-weaning. This positive effect could be associated to the characteristics of this type of ZnO, which has both a systemic and local effect. Hi is characterised by a slower release time, holding a different kinetic of Zn intestinal uptake which in turn has a local effect on the intestinal mucosa. In fact, Zn homoeostasis is regulated by a modulation of intestinal absorption efficiency (Dalto et al., 2023), that is inversely proportional to intake of Zn (Revy et al., 2003). A validation of the Zn source impact on the Zn absorption was also observed in the expression of the genes related to Zn transporter. Indeed, at d21 both the E and N piglets fed the SO_4_ tended to increase the expression of *SLC39A4*. This gene encodes for the ZIP4 transporter and is involved in the pathway of increasing intracellular Zn concentration. Actually, the expression of this protein (ZIP4) prevent the overload of Zn inside the enterocytes (Brugger et al., 2021). Indeed, an increase in ZIP4 expression has been associated with a Zn-deficient status in mice (Liuzzi et al., 2004). Moreover, it was confirmed in piglets that ZIP4 represent the major transporter to the enterocytes, playing an important role in the Zn homeostasis, and, particularly, its absorption. In fact, Brugger et al. (2021) observed an increase in Zn absorption correlated to a state of Zn deficiency. Therefore, it can be hypothesised that the administration of ZnSO_4_ was not as efficient as the porous form of ZnO in meeting the animals’ requirements.

## 5 Conclusion

As expected, weaning age was the most influential variable in the study. However, the observed differences in growth performance between early- and normal-weaned piglets were primarily attributed to physiological differences associated with their ages rather than the weaning age itself. Nevertheless, the porous form of ZnO and Zn-Gly, when compared to ZnSO₄, were able to improve small intestinal architecture, particularly during the post-weaning recovery phase. Additionally, the use of the porous form of ZnO, characterised by its high surface area and slower release rate, provides both systemic and local benefits, leading to a favourable gut microbiota composition, particularly increasing the relative abundance of beneficial Lactobacillaceae. In conclusion, the study demonstrated that Zn-Gly and porous ZnO represent favourable alternatives to ZnSO₄, particularly in early weaned piglets. While overall growth performance did not differ among Zn sources, the improvements in gut morphology, microbial balance, and potential antioxidant capacity observed with Zn-Gly and Hi underscore the importance of these Zn form for enhancing gut resilience and promoting long-term piglets’ health and productivity.

## Ethical approval and animal care

The in vivo trial was approved by the Ethics Committee for Experiments on Animals of the University of Bologna, Italy and by the Italian Ministry of Health (ID: 4401, 2216A.N.MCL, released 16th December 2022 in compliance with art. 31 of the D.lgs. 26/2014) and complied with the Animal Research Reporting of In Vivo Experiments (ARRIVE) guidelines.

## CRediT authorship contribution statement

**C. Negrini:** Writing – review & editing, Writing – original draft, Methodology, Investigation, Data curation. **D. Luise:** Writing – review & editing, Writing – original draft, Methodology, Investigation, Validation, Project administration, Supervision. **F. Correa:** Writing – review & editing, Methodology, Investigation, Data curation, Validation. **M. Mazzoni:** Writing – review & editing, Data curation, Investigation, Supervision. **A. Serra:** Writing – review & editing, Data curation, Investigation. **A. Monteiro:** Writing – review & editing, Conceptualization, Investigation. **P. Trevisi:** Writing – review & editing, Conceptualization, Investigation, Project administration, Supervision, Resources.

## Declaration of competing interest

The authors declare the following financial interests/personal relationships which may be considered as potential competing interests: Alessandra Monteiro is an Animine employers. If there are other authors, they declare that they have no known competing financial interests or personal relationships that could have appeared to influence the work reported in this paper.

## Data Availability

The raw reads obtained are publicly available at the NCBI Sequence Read Archive (SRA) under the accession number: PRJNA1289735.

## Appendix Supplementary materials

Image, application 1

Image, application 2
