# Supplementary material for: Effects of European authorised level of zinc from different sources on the physiology and intestinal ecosystem and performance of piglets weaned at different ages
Source: Vet Anim Sci. 2025 Oct 10;30:100518. doi: 10.1016/j.vas.2025.100518 (PMC12553058; doi:10.1016/j.vas.2025.100518)
Supplement: Supplementary file 2 [file mmc2.docx]

**Supplementary Table S1.** Composition of standard diet.

| Ingredients, % | | Calculated composition | |
| --- | --- | --- | --- |
| Corn and barley flaked | 20.00 | Dry matter (%) | 89.97 |
|  |  |  |  |
| Barley | 19.80 | Crude protein (%) | 19.00 |
| Wheat | 10.00 | Fat (%) | 5.48 |
| Wheat Middlings | 10.00 | Crude fibre (%) | 3.74 |
| Soybean Meal 48% crude protein | 7.00 | Ash (%) | 4.50 |
| Corn Extra Fine | 5.00 | NDF (%) | 14.10 |
| Soft Bran | 5.00 | ADF (%) | 4.60 |
| Extruded Rice | 5.00 | ADL (%) | 0.77 |
| Sweet Whey Spray | 4.50 | Starch (%) | 35.57 |
| Swine Plasma | 2.80 | Sugars (%) | 7.99 |
| Soy Oil | 2.00 | Metabolised energy, kcal/kg | 3283.00 |
| Soy protein concentrate | 1.50 | Ca (%) | 0.61 |
| Fiber Concentrate | 1.00 | P (%) | 0.72 |
| Potato protein concentrate | 1.00 | Avail. P (%) | 0.47 |
| Beet Pulp | 1.00 | Na (%) | 0.25 |
| Coconut Oil | 1.00 | K (%) | 0.82 |
| Aroma | 0.09 | Cl (%) | 0.51 |
| L-Lysine | 0.62 | Mg (%) | 0.12 |
| Dicalcium Phosphate | 0.60 | S (%) | 0.15 |
| Sodium Chloride | 0.40 | Fe (mg/kg) | 160.70 |
| Calcium Formate | 0.30 | Cu (mg/kg) | 106.00 |
| Formic Acid and Sodium Formate Buffered Solution | 0.30 | Zn (mg/kg) | 24.05 |
| Vitamin B Premix | 0.25 | Mg (mg/kg) | 84.38 |
| L-Threonin | 0.24 | Se (mg/kg) | 0.45 |
| DL-Methionine | 0.21 | Lysine SID (%) | 1.25 |
| Val-Ileu-Leu-His Premix | 0.20 | Methionine SID (%) | 0.40 |
| L-Triptophan | 0.05 | Threonine SID (%) | 0.27 |
| Iron Sulphate 21.5% | 0.05 | Tryptophan SID (%) | 0.81 |
| Vit A-D3-E 50-4-50 | 0.04 | Methionine + Cysteine SID (%) | 0.75 |
| Vit E 50% | 0.02 | Valine SID (%) | 0.85 |
| Copper Sulphate | 0.02 | Isoleucine SID (%) | 0.71 |
| Manganese Oxide | 0.01 | Leucine SID (%) | 1.36 |
| Potassium Iodide | 0.0001 | Histidine SID (%) | 0.45 |
| Zn Sulphate (40%) for SO_4_ group | 0.028 | Arginine SID (%) | 1.01 |
| Zn Glycinate (25%) for Gly group | 0.044 |  |  |
| Hizox (77%) for Hi group | 0.014 |  |  |

**Supplementary Table S2.** Real Time assay and probes.

| Target gene | Gene complete name | Assay Id | TaqMan® |
| --- | --- | --- | --- |
|  |  |  | Assay Catalogue |
| *NFKB2* | Nuclear Factor Kappa B Subunit 2 | Ss06883741_g1 | 4331182 |
| *GPX-2* | Glutathione Peroxidase 2 | Ss03387478_u1 | 4351372 |
| *SLC39A4* | Solute Carrier Family 39 Member 4 | Ss06910319_gH | 4351372 |
| *SLC30A7* | Solute Carrier Family 30 Member 7 | Ss03819782_s1 | 4351372 |
| *CLAUD4* | Claudin-4 | Ss03375006_u1 | 4351372 |
| *HMBS* | Hydroxymethylbilane Synthase | Ss03388782_g1 | 4351372 |

**Supplementary Table S3.** Effect of Zinc form and weaning age on performance parameters of Early and Normal piglets at the same physiological age.

| Item | Early | Normal | SEM | P-value | | |
| --- | --- | --- | --- | --- | --- | --- |
|  |  |  |  | Diet^1^ | Age^2^ | |
| Body weight, g | | | | | |  |
| Age 26-28 | 7037 | 8679 | 173.5 | 0.89 | <0.0001 | |
| Age 33-35 | 8521 | 9047 | 198.5 | 0.73 | 0.06 | |
| Age 40-42 | 11148 | 11419 | 336.5 | 0.85 | 0.56 | |
| Average daily gain, g/d | | | | | |  |
| Age 26-28 to 33-35 | 218.2 | 53.4 | 11.295 | 0.95 | <0.0001 | |
| Age 33-35 to 40-42 | 377 | 331 | 22.5 | 0.96 | 0.14 | |
| Feed intake, g/d | | | | | |  |
| Age 26-28 to 33-35 | 310 | 110 | 11.55 | 0.76 | <0.0001 | |
| Age 33-35 to 40-42 | 509 | 415 | 24.9 | 0.86 | 0.008 | |
| Gain to Feed | | | | | |  |
| Age 26-28 to 33-35 | 0.695 | 0.39 | 0.08 | 0.33 | 0.005 | |
| Age 33-35 to 40-42 | 0.74 | 0.79 | 0.03 | 0.53 | 0.19 | |

^1^SO_4_: ZnSO_4_, Gly: Zn-Glycinate, Hi: porous form of ZnO; ^2^Early: 21 days at weaning (6753.75±37 g), Normal: 26 days at weaning (9160±66 g).


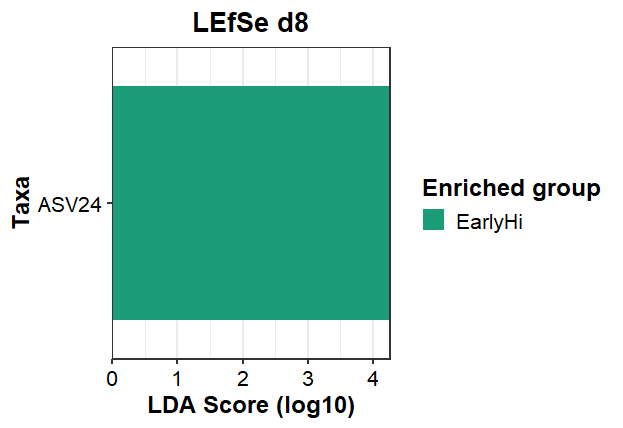

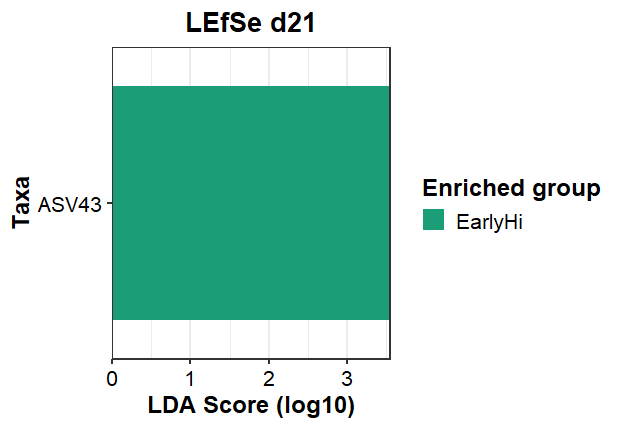


A

B

**Supplementary Figure S1**. Effect of Zinc administration and age at weaning category on taxa abundance at ASVs level in jejunum content samples of post-weaning piglets at d8 and d21 calculated on singular base. EarlyHi: porous form of ZnO, 21 days at weaning (6753.75±37 g).
